# QUALITY OF LIFE IN ADULTS LIVING WITH COGNITIVE IMPAIRMENT: DEVELOPMENT OF A UNIVERSAL MEASURE

**DOI:** 10.1093/geroni/igac059.654

**Published:** 2022-12-20

**Authors:** Keith Anderson, Lisa Peters-Beumer, Megan Westmore, Rebecca Logsdon

**Affiliations:** University of Mississippi, Oxford, Mississippi, United States; Concordia University Chicago, River Forest, Illinois, United States; University of Texas at Arlington, Arlington, Texas, United States; University of Washington, Seattle, Washington, United States

## Abstract

Quality of life is one of the most important and holistic measures of perceived overall well-being across the lifespan. For older adults living with cognitive impairment, quality of life has been conceptualized as consisting of an array of intertwined domains, such as relationship quality, physical health and ability, and opportunities for roles and activities. A subset of measures have been developed to gauge quality of life in older adults living with cognitive impairment, yet no “gold standard” in measurement has emerged. Quality of Life – Alzheimer’s Disease (QOL-AD) is one of the most commonly used and long-standing measures, yet it was developed in 2002 and has yet to be updated to reflect the evolution of our understanding of quality of life in adults living with cognitive impairment. Drawing from the literature on adults living with intellectual and developmental disabilities (IDD), the researchers identified several domains and items to update and expand the applicability of the QOL-AD measure. The proposed revised measure, Quality of Life – Alzheimer’s Disease/Intellectual and Developmental Disabilities (QOL-AD/IDD), is intended for adults living with cognitive impairment regardless of age or cause of impairment (e.g., dementia, IDD). The QOL-AD/IDD retains the format of the original measure, most notably the useful prompts that accompany each item and the supplemental proxy measure for caregivers. In this presentation, the researchers will discuss the development of the QOL-AD/IDD and ongoing (e.g., Delphi review) and planned steps to evaluate the reliability and validity of this promising measure.

